# Cognitive abilities and physical activity in chronic kidney disease patients undergoing hemodialysis

**DOI:** 10.1590/1980-57642018dn13-030010

**Published:** 2019

**Authors:** Raiana Lídice Mór Fukushima, Pollyanna Natalia Micali, Elisangela Gisele do Carmo, Fabiana de Souza Orlandi, José Luiz Riani Costa

**Affiliations:** 1 Universidade Estadual Paulista Ringgold standard institution - Physical Education Rio ClaroSP Brazil Universidade Estadual Paulista, Ringgold standard institution - Physical Education, Rio Claro, São Paulo, SP, Brazil.; 2 Universidade Estadual Paulista Julio de Mesquita Filho Ringgold standard institution - Educação Física Rio ClaroSP Brazil Universidade Estadual Paulista Julio de Mesquita Filho Ringgold standard institution - Educação Física Rio Claro, São Paulo, SP, Brazil.; 3 Universidade Federal de São Paulo Ringgold standard institution Sao CarlosSP Brazil Universidade Federal de São Paulo, Ringgold standard institution, Sao Carlos, SP, Brazil.; 4 Universidade Estadual Paulista Julio de Mesquita Filho Instituto de Biociências Ringgold standard institution - Instituto de Biociências Rio ClaroSP Brazil Universidade Estadual Paulista Julio de Mesquita Filho Instituto de Biociências Campus de Rio Claro Ringgold standard institution - Instituto de Biociências, Rio Claro, SP, Brazil.

**Keywords:** mental health, renal insufficiency, exercise, health, quality of life, saúde mental, insuficiência renal, exercício, saúde, qualidade de vida

## Abstract

Hemodialysis (HD) is a common treatment for Chronic Kidney Disease (CKD).

**Objective:**

the objective of the present study was twofold. First, to assess the level of physical activity and cognitive function in hemodialysis (HD) patients and, second, to compare cognitive function of active and insufficiently active HD patients.

**Methods:**

the sample consisted of 84 HD patients. Data collection took place in two different Renal Replacement Therapy Units (RRTU) in Brazil. A sociodemographic and clinical structured interview, the International Physical Activity Questionnaire (IPAQ) and the Addenbrooke's Cognitive Examination Revised (ACE-R) were used for data collection. The Mann Whitney U-test was used to compare cognitive function between groups. The significance level adopted was p≤.05.

**Results:**

the majority of the patients were insufficiently active. The total average score on the cognitive test was lower than recommended and physically active HD patients had a significant score in the fluency domain.

**Conclusion:**

we found that HD patients are at greater risk of developing cognitive deficits. Physical activity was shown to be a potential non-pharmacological, low-cost therapeutic alternative for improving cognitive abilities in HD patients. The present study can help health professionals to encourage HD patients to engage in regular physical activity and contributes to the development of specific protocols for these patients.

## RESUMO

A hemodiálise (HD) é um tratamento frequente para a Doença Renal Crônica (DRC).

**Objetivo:**

primeiro, avaliar o nível de atividade física e função cognitiva de pacientes em hemodiálise (HD) e, segundo, comparar a função cognitiva entre pacientes ativos e insuficientemente ativos em HD.

**Métodos:**

84 pacientes em HD participaram do estudo. A coleta de dados ocorreu em duas diferentes Unidades de Terapia Renal Substitutiva (UTRS) no Brasil. Uma entrevista sociodemográfica e clínica estruturada, o Questionário Internacional de Atividade Física (IPAQ) e o Exame Cognitivo de Addenbrooke Revisado (ACE-R) foram utilizados para coleta de dados. O teste U de Mann Whitney foi usado para comparar a função cognitiva entre os grupos. O nível de significância adotado foi p≤0,05.

**Resultados:**

a maioria dos pacientes apresentou-se insuficientemente ativa. A pontuação média total do teste cognitivo foi menor do que a recomendada e os pacientes em HD fisicamente ativos obtiveram um escore significativo no domínio da fluência verbal.

**Conclusão:**

os pacientes em HD podem apresentar um maior risco para desenvolver déficits cognitivos. A atividade física demonstrou ser uma alternativa terapêutica não farmacológica e de baixo custo para otimizar a função cognitiva em pacientes em HD. Espera-se que a presente pesquisa ajude os profissionais de saúde a incentivar os pacientes em HD a praticar regularmente a atividade física e contribuir para o desenvolvimento de protocolos específicos para esses pacientes.

Hemodialysis (HD) is a common treatment for Chronic Kidney Disease (CKD).^1^ The Kidney Disease Improving Global Outcomes (KDIGO) defined CKD as abnormalities of kidney structure or function, present for a period over three months, and with implications for health.^1^ According to the Brazilian Dialysis Survey, the estimated number of dialysis patients in 2016 was 122,825. This figure represents an increase of 31,511 patients within the last 5 years (91,314 in 2011), and an annual growth of 6.3% in the number of patients.^2^

A number of authors have shown that CKD patients have a higher risk of developing dementia.^3^^-^5 Dementia represents a loss of cognitive functioning sufficiently severe to compromise several cognitive abilities, but not limited to attention/concentration, executive functions, memory, logical reasoning, visual-spatial ability, language and judgement.^6^^,^^7^

Dementia may account for worsening of CKD side effects (i.e. disability, hospitalization, dialysis withdrawal and mortality),^8^ and there is sound evidence that both diseases represent key factors in compromising quality of life (QOL). Furthermore, declines in cognition are associated with increased risks of comorbidity and mortality.^9^^,^^10^

Studies have shown that increased serum cystatin C and albuminuria are also associated with accelerated cognitive decline, and there is a 15-25% increased risk of cognitive deficit for every 10 ml/min per 1.73 m^2^ reduction in estimated glomerular filtration rate (eGFR). Furthermore, there is an increased odds ratio of 2.43 (95% CI, 1.38 to 4.29) for cognitive deficits in patients with an eGRF of <45 ml/min per 1.73 m^2^, even after adjusting for possible confounders.^11^^,^^12^ Another postulated theory for loss of cognitive functioning in CKD patients is that these patients often have underlying vascular disease such as diabetes mellitus and hypertension, which may contribute to cognitive impairment and, consequently, exert a negative influence on QOL.^13^

In contrast, non-pharmacological treatments such as physical activity may positively contribute to cognitive functioning in CKD patients.^13^ The physiological mechanisms underlying this improved cognition resulting from physical activity may include: 1) increased oxygen and nutrients supply for cerebral metabolism due to an increase in cerebral blood flow; 2) increased neurotransmitter expression capable of altering action potentials; and 3) modulation of several hormones (i.e. β-endorphin, cortisol), which may affect the morphology of brain structures associated with cognitive functions.^13^

However, recent scientific evidence elucidating the influence of physical activity on cognitive function in the Brazilian HD population is scarce. Therefore, the objective of the present study was twofold. First, to assess the level of physical activity and cognitive function in HD patients and, second, to compare cognitive function of active and insufficiently active HD patients.

## METHODS

The present study had a descriptive, cross-sectional, quantitative design. It was conducted in two different Renal Replacement Therapy Units (RRTU) in Brazil. Convenience sampling was adopted, which consisted of a non-probability sampling method. The following inclusion criteria were used: 1) patients diagnosed with CKD; 2) aged 18 or older; 3) patients who had been on HD for at least three months. Thus, the sample comprised 84 patients.

Three questionnaires were applied for data collection. The first questionnaire collected sociodemographic (gender, age, and education) and clinical (hemodialysis time, underlying diseases and medications use) data. Regarding the sociodemographic and clinical data, there was a predominance of men (69%), mean age of 52.6 (±14.3) years, with education up to complete high school (52.4%), average HD time of 39.2 (±50.3) months, diabetes type I and II (4.8%), hypertension (28.6%), and both diabetes and hypertension (27.4%) were most prevalent underlying diseases, and 92.9% used medications. The Addenbrooke's Cognitive Examination Revised (ACE-R), a brief cognitive battery for dementia screening was then applied. The ACE-R was developed in 2006.^14^ It was translated and adapted for use in the Brazilian context,^15^^,^^16^ and due to its high sensitivity and specificity, the ACE-R has proven to be useful for detecting early cognitive dysfunction, differentiating between AD and frontotemporal dementia, as well as effectively evaluating several commonly affected cognitive functions in the initial stage of the disease.^16^ The ACE-R evaluates five cognitive domains: attention (e.g. orientation; registration of three items and serial 7 subtraction) (18 points), memory, (e.g. recall of three items; anterograde memory and retrograde memory) (26 points), fluency (e.g. create as many words as possible beginning with the letter “P” and name as many animals as possible with any letter) (14 points), language (e.g. comprehension; sentence writing; single word repetition; object naming and reading) (26 points) and visuospatial abilities (e.g. copy a pentagon and 3-D wire cube; counting dots without using hands; identifying letters) (16 points).^16^ In the present adaptation of the battery, the Brazilian version of the Mini-Mental State Examination (MMSE) was used, therefore, some tasks were kept the same as the MMSE.^16^ ACE-R scores range from zero to 100 points, and the cut-off point <78 demonstrated high sensitivity and specificity (100.0% and 82.3%, respectively) for the diagnosis of mild AD.^15^^,^^16^ The third questionnaire was the International Physical Activity Questionnaire (IPAQ). The IPAQ was an International Consensus Group effort to develop a measure of physical activity suitable for assessing levels of physical activity worldwide. There are two versions of the questionnaire - short and long versions - and, in order to have access to the amount of time spent on physical activity for each domain, we opted for the long version,^17^ whereas in the short version, the outcome is only a total score for physical activity.^17^ The IPAQ was divided into four dimensions (work, transportation, household and leisure). In each domain, the number of minutes spent on both moderate and vigorous physical activity that extended for a minimum of 10 minutes was recorded. According to the procedure of Hallal et al.,^18^ the total physical activity score was quantified as the sum of the number of minutes of moderate activity plus two times the number of minutes of vigorous activity for each of the four domains. For analysis purposes, insufficient physical activity was defined in accordance with the World Health Organization (WHO) guidelines. Therefore, HD patients who reported ≥ 150 minutes of combined moderate and vigorous physical activity per week were considered active. Similarly, HD patients who reported <150 minutes of combined moderate and vigorous physical activity per week were considered insufficiently active. The validity and reproducibility of the IPAQ for the Brazilian population was determined by Matsudo et al.^19^

Informed consent was obtained from all participants who agreed to participate in the study, as established by the Brazilian National Health Council.^20^ Interviews were performed individually in a private setting by a single evaluator, which allowed homogeneity of results. All procedures performed in studies involving human participants were in compliance with the ethical standards of the institutional and/or national research committee and with the 1964 Helsinki declaration and its later amendments or comparable ethical standards. The present study was approved by the Ethics Committee of São Paulo State University, under permit number 1.537.827.

All statistical treatment was performed using the Statistical Package for the Social Sciences (SPSS). The quantitative analysis was treated using descriptive statistics including measures of frequency (percentage), central tendency (mean) and dispersions (standard deviation). In addition, the Kolmogorov-Smirnov test was used to verify the lack of normal distribution. Thus, the non-parametric Mann Whitney U-test was used to compare the cognitive function between groups (active and insufficiently active). The significance level adopted for the statistical tests was 5% (p≤.05).

## RESULTS

Table 1 shows the prevalence of active and insufficiently active HD patients on each domain of the IPAQ. A high prevalence of insufficiently active HD patients was evident for all four domains, indicating a lower engagement of HD patients in physical activity.

**Table 1:** Prevalence of active and insufficiently active HD patients, by domain.

Domains	Active (%)	Insufficiently active (%)
Work	7.1	92.8
Transportation	26.2	73.8
Household	42.8	57.1
Leisure	16.7	83.3

Table 2 shows mean scores, standard deviation and cut-off points of the ACE-R. The average mean scores of the ACE-R did not surpass the cut-off points on any of the domains, including total points, indicating HD patients may be at a greater risk of developing cognitive deficits.

**Table 2:** ACE-R descriptive data, by domain.

Domains	n	Mean	SD*	Cut-off point	% n (below cut-off point)**
Attention	84	15.9	2.1	<17 points	67.9
Memory	84	15.5	2.8	<15 points	44.0
Fluency	84	5.9	3.3	<8 points	74.0
Language	84	20.5	6.5	<22 points	46.4
Visuospatial	84	10.8	4.1	<13 points	63.1
Total	84	68.6	14.5	<78 points	74.0

* Standard deviation.

** Percentage of patients with mean score below the cut-off point.

Table 3 provides a comparison of active and insufficiently active HD patients. There was a slight difference in cognitive assessment scores between active and insufficiently active HD patients. Interestingly, active HD patients had a statistically significant higher score (p-value <.005) on the fluency domain.

**Table 3:** Comparison of active and insufficiently active HD patients and cognitive assessment.

Domains	n	Active (n = 51)	Insufficiently active (n = 32)	p-value
Attention	84	15.8	16.0	0.602
Memory	84	15.5	15.5	0.562
Fluency	84	6.8	4.8	0.005
Language	84	20.4	20.6	0.842
Visuospatial	84	11.2	10.2	0.390
Total	84	69.7	67.0	0.405

## DISCUSSION

The diagnosis of a chronic, progressive, irreversible disease (i.e. CKD) that requires complex treatment (i.e. HD) is associated with a variety of complications for daily life. Although HD treatment is capable of slowing CKD progression to end-stage renal disease (ESRD), it can be exhausting treatment. Clinical complications such as pain, cramps, nausea, vomiting, diarrhea, dyspnea, together with possible side effects of the medications used to stabilize these symptoms, can negatively influence QOL.^21^ Additionally, CKD may be a risk factor for cognitive impairment. However, both CKD and cognitive impairment may be alleviated through healthy choices, such as engaging in regular physical activity. Therefore, to assess and compare levels of physical activity and cognitive function among HD patients may represent the starting point for health professionals in tackling the rising incidence of CKD patients and, consequently, the incidence of dementia.

In the current study, the majority of patients were classified as insufficiently active, according to the 2011 WHO guidelines (<150 minutes per week) (Table 1). The current findings are similar to those of a study of HD patients, in which 74.5% of the sample was either sedentary or insufficiently active.^22^ Furthermore, a 7-year-follow-up retrospective cohort study observed that, in order to improve HD patient prognosis, one of the requirements was to prevent decline in physical activity over time.^23^ A recent investigation concluded that patients who did not engage in any type of physical activity had faster onset of CKD compared to a sample that exercised regularly.^24^ Another study showed that physical activity was directly associated with an increased QOL and reduced morbidity and mortality, both in the general population and CKD patients.^25^^-^27

It has been suggested that muscle weakness is a frequent complication of CKD,^28^ although its etiology has not been fully elucidated. However, several authors have cited some risks factors for muscle weakening, such as carnitine deficiency, malnutrition, myopathy, muscular atrophy, parathyroid hormone (PTH) excess and toxicity, uremic toxins and vitamin D deficiency.^28^^,^^29^ There is also atrophy of both types of fibers, mainly type II fibers.^28^ These findings may explain the low adherence to engagement in physical activity. Conversely, another study noted that physical exercise can contribute to normal muscle tension and venous return, attenuating the rapid fluid loss promoted by HD.^29^ Regular physical activity benefits cardiometabolic and metabolic functions across all stages, providing an approach to address common comorbidities in CKD patients. Also, the maintenance of muscle health is related to renoprotective effects.^27^ Therefore, it is possible to reinforce the importance of multiple types of exercises to minimize loss of muscle mass, as well as to promote the strength necessary for individuals to perform activities of daily living (ADLs) with less effort.^30^

Table 2 shows that the total average score (ACE-R) was lower than the cut-off points (78 points),^15^^,^^16^ confirming HD patients are at greater risk of developing dementia.^5^^,^^7^^-^13 Cognitive symptoms such as memory disorders, difficulty planning activities, impaired attention, decreased information processing speed, motor disability, and speech difficulty have been shown in mental health and CKD, revealing a negative influence of CKD on mental fitness.^31^

As observed in Table 3, insufficiently active patients had equal or lower average scores, suggesting that a higher level of physical activity may contribute to a higher ACE-R score in the studied population. Similar findings were observed in the literature, where authors reported that physically active patients attained better cognitive test scores (30). Physical training can induce positive changes in brain metabolism.^31^ There has been speculation that exercise may promote adaptations in brain structures and synaptic plasticity, culminating in cognitive improvement.^32^ Thus, exercise training programs appear to be effective and safe for the CKD population.^32^^-^34

Finally, physically active patients had a statistically significant score (p-value <.005) on the fluency domain (Table 3). It is important to mention that education plays a crucial role in determining performance on cognitive tests. Another possible variable that might have been related to the statistically significant score in the verbal fluency domain was age. A relationship was revealed among cognitive abilities and age, education and HD time, suggesting that HD patients who were older, had less education and longer HD time had greater cognitive deficits.^35^ According to an investigation in 2014, verbal fluency tasks seemed to be differentially affected by age and education.^36^ The repeated measures analysis revealed an age effect on the semantic verbal fluency task, whereas education affected performance on phonemic and semantic verbal fluency tasks. The ACE-R has two demanding tasks, which might have proven more challenging for those with modest cognitive acquisition. However, as previously mentioned, the educational level of the sample was up to complete high school and mean age was 52.6 (±14.3) years, providing a possible explanation for the statistically significant score on only one domain (e.g. verbal fluency).

This result corroborates an earlier investigation which found that HD patients had worse performance than controls on tests evaluating logical reasoning, verbal learning, motor ability, verbal fluency and visuospatial memory.^34^ A systematic review showed that cognitive changes occur early in CKD patients and at a much faster rate than in the general population. Orientation, attention and language are particularly affected.^37^ Additionally, dialyzed patients produced fewer words on a phonemic fluency task and the performance of these patients significantly declined over a period of approximately two years.^38^ However, a possible relationship was suggested between physical activity and cognitive function, albeit weak, where results indicated superior performance on variables of the verbal fluency task among physically active elderly.^39^

The present study has a number of limitations. First, the small sample size and the fact that data was collected at only two RRTU. Second, the difference between active and insufficiently active HD patients cannot be explained by level of physical activity alone. Therefore, future studies should control for possible confounders. Finally, the cross-sectional design precluded the establishment of a cause-and-effect relationship. Similarly, several strengths should be mentioned. The results confirmed HD patients are at greater risk of developing cognitive deficits. Physical activity was shown to be a potential non-pharmacological and low-cost therapeutic alternative for improving cognitive abilities in HD patients. Therefore, while the present study supports that physical activity may reduce cognitive decline in HD patients, the clinical significance of these findings needs to be established.

In conclusion, the present study can help health professionals encourage HD patients to engage in regular physical activity and contribute to the development of specific protocols for these patients. The findings highlight the importance of boosting physical activity as an alternative to promote mental health or the maintenance of cognitive skills essential for the independence of HD patients. Nonetheless, further recent scientific evidence of the benefits of physical activity to avert cognitive decline is needed to inform and raise awareness of Brazilian researchers and health professionals in an effort to attenuate the rising incidence of CKD and cognitive decline, and to provide psychosocial support and education on crucial topics (e.g. control of blood pressure), thereby improving overall quality of life of this patient group.
